# Anal Cancer Screening: 10-Year Experience of a Specialized Outpatient Clinic

**DOI:** 10.3390/cancers17020193

**Published:** 2025-01-09

**Authors:** Iolanda Espirito Santo, Amaniel Kefleyesus, Camille Chilou, Seraina Faes, Daniel Clerc, Martin Hübner, Dieter Hahnloser, Fabian Grass

**Affiliations:** 1 Department of Visceral Surgery, Lausanne University Hospital (CHUV), 1011 Lausanne, Switzerland; iolanda.espiritosanto@distalmotion.com (I.E.S.); amaniel.kefleyesus@chuv.ch (A.K.); dieter.hahnloser@chuv.ch (D.H.); 2 Faculty of Biology and Medicine (FBM), University of Lausanne (UNIL), Rue du Bugnon 21, 1011 Lausanne, Switzerland; 3 Stadtspital Triemli, 8063 Zürich, Switzerland; 4 Hôpital de Sion, 1951 Sion, Switzerland

**Keywords:** anal cancer, high-resolution anoscopy, HPV testing, anal dysplasia, screening, algorithm, HIV, MSM

## Abstract

In 2012, the Department of Visceral Surgery of the Lausanne University Hospital CHUV implemented a dedicated high-resolution anoscopy (HRA) outpatient clinic for surveillance and follow-up purposes. This 10-year longitudinal study analyzed 537 patients (2214 visits) using a structured screening protocol. Dysplastic lesions were detected in 49% of patients, predominantly low-grade squamous intraepithelial lesions (LSILs, 74%). Among LSIL cases, 6% progressed to high-grade squamous intraepithelial lesions (HSILs) within 24 months, reaching 25% cumulative progression at 36 months. Of HSIL patients, 3% developed carcinoma in situ after 48 months. Notably, no invasive carcinoma was observed during the follow-up. Four patients diagnosed with squamous cell carcinoma at initial screening were treated with chemoradiotherapy, and one required salvage surgery. Independent risk factors for the presence of higher-stage precancerous lesions (≥HSILs) were the presence of high-risk HPV genotypes (OR 14.5, 95% CI 5–42.2, *p* < 0.001), detectable HIV viral load (OR 5.4, 95% CI 1.8–16.7, *p* = 0.003), and symptoms at the first screening visit (OR 3.2, 95% CI 1.1–9.9, *p* = 0.04). HIV-positive status was associated with a trend towards an increased risk of progression (OR 2.79, *p* = 0.073). These findings highlight the importance of systematic follow-up and early intervention in high-risk populations to prevent anal cancer progression.

## Simple Summary

The incidence and mortality of anal cancer has risen in recent years. This longitudinal study over a ten-year period analyzed 2214 outpatient visits of 537 patients using high-resolution anoscopy (HRA)-based screening and standardized surveillance and treatment protocols. Dysplastic lesions were revealed in 49% of patients. While no invasive carcinomas developed during a median follow-up period of 48 months, 3% of HSIL patients progressed to carcinoma in situ. HIV-positive status showed an almost three-fold increased progression risk. The present study highlights the importance of dedicated screening, close surveillance, and early intervention in high-risk groups to reduce the risk of disease progression to anal cancer.

## 1. Introduction

Anal cancer represents only 3% of all gastrointestinal tumors [1]. However, its incidence has consistently increased over the past five decades, reaching 0.5–2.0/100,000 per year in 2017 [2,3]. While the highest incidence is observed around age 65, recent data indicate a worrying rise among the younger, sexually active population [4]. Interestingly, age-standardized incidence is highest in Switzerland among European countries according to a recent epidemiologic study [5].

The pathophysiology of anal cancer is thought to be associated with a chronic inflammatory state [6]. From an anatomy point of view, cancers of the anal margin (harboring squamous epithelium) and anal canal (harboring cylindrical epithelium) must be considered as separate entities with distinguished histopathological features, patterns of local invasion, and lymphatic drainage, with squamous cell carcinoma (SCC) representing the vast majority of anal cancers [7]. Human papillomavirus (HPV) is the primary etiological factor, with high-risk genotypes 16 and 18 detected in up to 90% of precursor lesions and cancers [8]. In line with the pathogenesis of cervical cancer, the virus is integrated into the host genome, inducing precursor lesions. The double-stranded DNA virus primarily infects and replicates within basal cells of transformation zones (squamous to columnar epithelium) and thus also has a tropism for the dentate line of the anus [9]. Microtears or abrasions in the epithelium are common origins for exposure [10,11]. Failure of several control mechanisms favorizes the progression of HPV-infected cells to low-grade squamous intraepithelial lesions (LSILs), high-grade squamous intraepithelial lesions (HSILs), carcinoma in situ, and invasive cancer. These mechanisms include intracellular control, likely mediated by cyclin-dependent kinase inhibitors, a paracrine signaling cascade, and immunological control mechanisms [12]. The dysplasia to cancer sequence usually takes several years, fostering a window of opportunity for surveillance and prevention strategies [13].

Human immunodeficiency virus (HIV) and the practice of receptive anal intercourse, in particular in men who have sex with men (MSM), represent two major risk factors, with a combined multi-fold increased risk of anal cancer [14,15]. Surveillance is thus crucial in populations at risk not only to prevent disease progression but also to provide education about the disease, highlight modifiable risk factors, and emphasize the importance of annual screening visits for individuals at risk.

Dysplastic lesions can be divided into two groups, according to histological criteria (nuclear size and membrane regularity, mitotic activity, cell differentiation, and depth of epithelium involvement), as (1) LSILs considered condyloma-like lesions and (2) HSILs considered and managed as precancerous lesions [10,16]. A consensus has yet to be found regarding optimal screening intervals for the surveillance of risk populations.

The surveillance of precancerous lesions and patient education are of utmost importance for anal cancer prevention given its association with HPV in up to 90% of patients, its association with modifiable risk factors such as sexual practices, and, most importantly, its pathognomonic dysplasia to cancer sequence [5]. In 2012, the Department of Visceral Surgery of the Lausanne University Hospital CHUV standardized surveillance and treatment by the launch of a dedicated outpatient surveillance clinic. The aim of this study was to perform a longitudinal analysis 10 years after its implementation to evaluate the effectiveness of the institutional screening and treatment protocols in a specialized setting while evaluating the risk of disease progression to cancer.

This study was presented at the Annual Meeting of the Swiss College of Surgeons, Davos, Switzerland, 30 May 2024, the Annual Meeting of the European Society of Coloproctology, Thessaloniki, Greece, 24 September 2024, and the European Colorectal Congress, St. Gallen, Switzerland, 2 December 2024.

## 2. Materials and Methods

### 2.1. Study Setting

This monocentric retrospective cohort study was conducted at Lausanne University Hospital (CHUV) between January 2013 and December 2022. Weekly consultations were performed at the dedicated high-resolution anoscopy (HRA) outpatient clinic, established in 2012 and led by board-certified colorectal surgeons supervising clinical fellows for educational purposes. This study did not involve any specific intervention beyond standard care practices for HRA screening and follow-up. Patient data were retrospectively analyzed to assess the outcomes of this screening program. In accordance with the Declaration of Helsinki, this study was approved by the institutional review board (CER-VD # 2022.00925), with all patients having provided written informed consent during their clinical care.

### 2.2. Patients

Patients aged ≥18 years were included if they presented with risk factors for anal dysplasia, such as HIV infection, positive cervical or anal HPV cytology, biopsy-proven squamous intraepithelial lesions (SILs), a history of dysplastic cervical or anal lesions, or non-HIV-related immunosuppression (e.g., transplant recipients). Patients with incomplete data, insufficient follow-up, or without documented consent for the use of their data were excluded.

### 2.3. Screening and Treatment Protocols

All patients underwent clinical examination, peri- and endoanal HRA screening, swabs and biopsies if warranted, and further treatments if indicated. HPV genotyping was routinely performed on biopsied tissue samples as part of the standard diagnostic workflow, particularly for high-grade lesions (HSILs). Prior results of HPV genotyping (before the study period) were also registered when available. Suspicious condyloma-like lesions were biopsied and sent to the institutional pathology lab for further analysis. HPV tests were performed with the MagNaPure 96 Total Nucleic Acids kit (Roche, Basel, Switzerland), with genotyping of 28 HPV genotypes (6, 11, 16, 18, 26, 31, 33, 35, 39, 40, 42–45, 51–54, 56, 58, 59, 61, 66, 68–70, 73, and 82) by the Anyplex™ II HPV28 kit (Seegene, Seoul, Republic of Korea). For the purpose of this study, the predominant high-risk (HR) HPV types 16 and 18 and low-risk (LR) HPV types 6 and 11 were specifically analyzed. Excisional biopsy of small condyloma-like lesions was performed under local anesthesia as the first treatment option. Cryosurgery was offered at the clinic in 3 bi-weekly intervals. Patients presenting with extensive or numerous lesions were referred to the institutional outpatient surgery facility for CO2 laser ablation and/or surgical excision under anesthesia. Topical treatments (imiquimod, 5-fluorouracil) were used for the treatment of recurrent localized lesions.

The screening protocols were based on standardized guidelines and adapted for logistic feasibility purposes to our institution [1]. Screening followed a previously established institutional treatment algorithm considering the available treatment modalities [17]. Subsequent follow-up intervals were scheduled according to the histopathological findings and risk profiles: patients with LSILs were followed-up every 6 months, while patients with more severe dysplastic lesions followed 3-month surveillance intervals. Patients without abnormal findings but with the risk factors specified above were followed-up by means of annual screening visits. During follow-up of a specific patient, new or suspicious lesions were biopsied to determine potential disease recurrence or progression.

### 2.4. Data Collection and Management

Baseline demographic data, including the Charlson Comorbidity Index [18], immunosuppression status (HIV- and non-HIV-related immunosuppression, i.e., transplant patients), HIV status including acquired immunodeficiency syndrome (AIDS) status, and HIV viral load according to standardized definitions [19], were determined. HIV status was routinely assessed through serological testing. Furthermore, sexual behavior (MSM), symptoms at the initial screening visit (i.e., burning, itching, bleeding), histopathological findings (carried out by the institutional department of pathology), and specific past (HPV-related history) and present treatments as specified above were prospectively recorded for all patients in a dedicated database.

Data were managed by clinical fellows and institutional data management resources. The initial stage of the lesion and potential progression during follow-up were documented to ascertain the effectiveness of the algorithm and estimate optimal follow-up intervals. Loss to follow-up was recorded, and end of follow-up was determined according to the available data during the study period.

### 2.5. Statistical Analysis

Sample characteristics were outlined using descriptive statistics. Continuous variables were represented as mean ± standard deviation or median and range, while categorical variables were summarized as frequencies and percentages.

Univariate tests were used to compare groups, with the Chi-square test for qualitative variables and Student’s *t*-test or the Mann–Whitney test for quantitative variables, depending on the data distribution. A one-way ANOVA or the Kruskal–Wallis test were used for multiple group comparisons, depending on the data distribution. Uni- and multivariable logistic regression were performed to calculate odds ratios (ORs), with 95% confidence intervals (CIs) for the presence of higher-stage (≥HSILs) lesions.

In the multivariable logistic regression model, risk factors were selected based on their clinical relevance and statistical significance in the univariable analysis (*p* < 0.1). The included variables were age ≥ 45 years, loss to follow-up, presence of HR HPV genotypes, Charlson Comorbidity Index ≥ 2, men who have sex with men (MSM), detectable HIV viral load, symptoms at the first screening visit, and female gender. These factors were analyzed to identify independent predictors of higher-stage precancerous lesions (≥HSILs). Cumulative risk analysis was conducted to quantify the association between risk factors and the transformation of low-grade lesions (LSILs) into high-grade lesions (HSILs) or carcinoma in situ, with ORs and 95% CIs.

All analyses were conducted using Jamovi software (version 2.4.5; The Jamovi Project, Sydney, Australia), SPSS Advanced Statistics 29 (IBM Software Group, Chicago, IL, USA), and GraphPad Prism Version 10.1.2. All tests were two-sided, and an alpha-level of 0.05 or less was considered statistically significant.

## 3. Results

### 3.1. Referrals and Demographics

**Figure 1:**
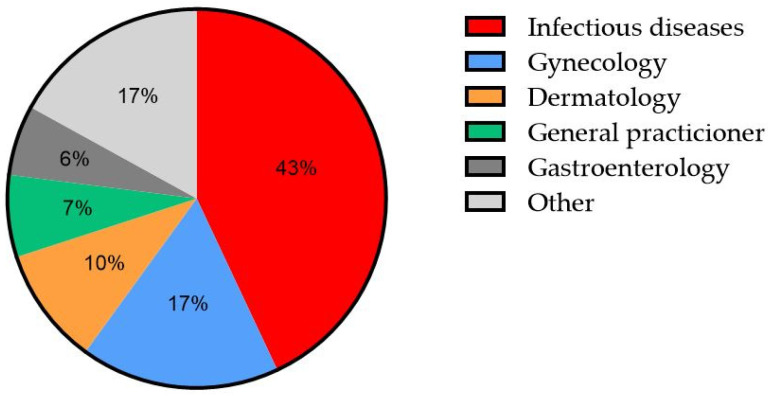
Patient referrals to the institutional HRA clinic. HRA—high-resolution anoscopy. Others include referring surgeons and local emergency departments.

Patients were referred from different care providers, mainly infectious disease specialists, gynecology, and dermatology, as outlined in Figure 1.

The study cohort consisted of 537 patients (70.2% male), with a median age of 47 years (IQR 38–57), totaling 2214 consultations over the study period. More than half (52.1%) tested positive for HIV, and more than two-thirds of male patients (70.2%) were MSM. The median length of follow-up was 51.4 months.

About half of the patients (51%) did not present any lesions during follow-up, while 263 (49%) presented with dysplastic lesions, mainly LSILs (74% of all lesions). Four patients presented with invasive squamous cell carcinoma (SSC), all of which were diagnosed at the initial screening visit.

Table 1 summarizes baseline demographics, immunosuppression status, and past treatments of the cohort.

### 3.2. Risk Factors for Higher-Stage Lesions

**Figure 2:**
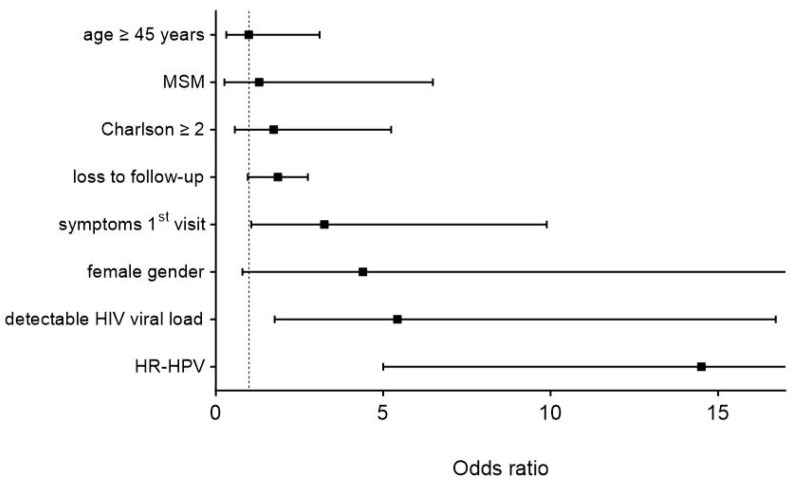
Multinominal logistic regression analysis of risk factors for higher-stage lesions (≥HSILs). Displayed are odds ratios (black squares) with 95% confidence intervals (black bars). Two error bars are clipped at the axis limit. Charlson—Charlson Comorbidity Index, MSM—men who have sex with men, HIV—human immunodeficiency virus, HR-HPV—presence of high-risk human papillomavirus genotypes.

Independent risk factors for the presence of higher-stage precancerous lesions (≥HSILs) were the presence of high-risk HPV genotypes (OR 14.5, 95% CI 5–42.2, *p* < 0.001), detectable HIV viral load (OR 5.4, 95% CI 1.8–16.7, *p* = 0.003), and symptoms at the first screening visit (OR 3.2, 95% CI 1.1–9.9, *p* = 0.04). Statistical trends were observed for the female gender (OR 4.4, 95% CI 0.8–24.1, *p* = 0.088) and loss to follow-up (OR 2.8, 95% CI 1–7.9, *p* = 0.059), as illustrated in Figure 2.

### 3.3. Treatment and Follow-Up

A total of 1114 procedures were performed, mainly three sessions of cryotherapy (43.4%), exams under anesthesia with excisional surgical and/or CO2 laser treatment (30.0%), and excisional biopsies under local anesthesia in the clinic (17.6%). Excisional biopsy represented the initial treatment in both HSIL (49%) and LSIL (32%) patients in order to determine a baseline diagnosis. The two patients presenting with in situ carcinoma during follow-up were treated by surgical R0 excision (safe margins), with no recurrence observed until study closure. All four patients with SCC were treated by exclusive chemoradiotherapy. One of these required subsequent salvage abdominoperineal resection for local recurrence 16 months later.

Loss to follow-up was observed in 170 patients (31.7%) overall. Among these, 33.1% were in the LSIL group, 37.8% in the ≥HSIL group, and 28.8% in the control group. The difference in the proportions across groups was not statistically significant (*p* = 0.35).

**Figure 3:**
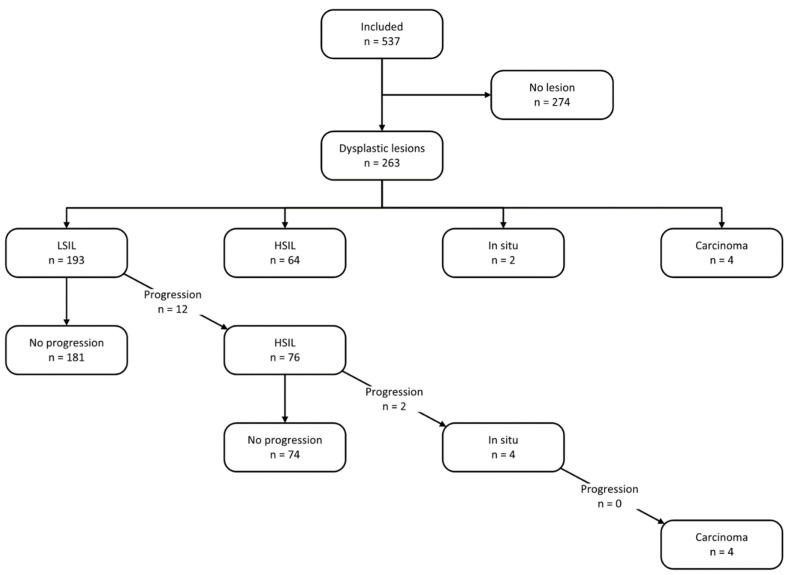
Histopathological findings during the first screening visit and progression rates during follow-up. LSIL—low-grade squamous intraepithelial lesion; HSIL—high-grade squamous intraepithelial lesion.

Figure 3 outlines histopathological findings and disease progression during follow-up.

**Figure 4:**
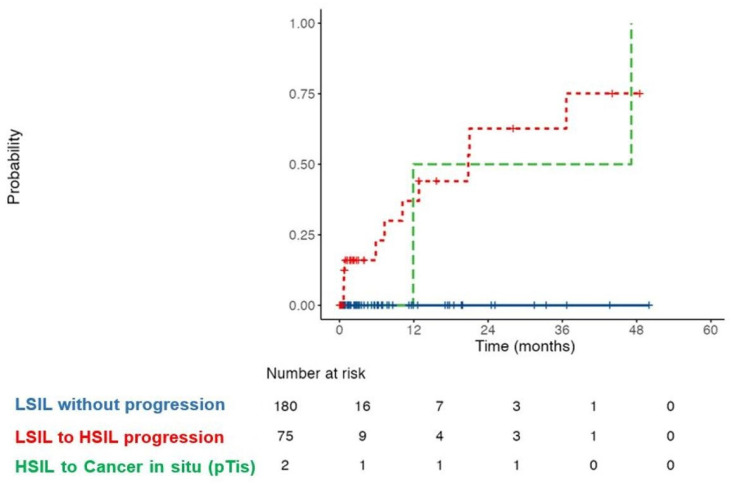
Cumulative risk of disease progression from LSIL to HSIL (red) and from HSIL to carcinoma in situ (green). LSIL—low-grade squamous intraepithelial lesion; HSIL—high-grade squamous intraepithelial lesion.

Over the study period, 6% (n = 12/193) of patients with LSILs progressed to HSILs with a median follow-up of 24 months. The cumulative probability of progression to HSILs at 12, 24, and 36 months was 10%, 18%, and 25%, respectively, while 37.3% of these patients progressed after 36 months of follow-up. In total, 3% of HSIL patients progressed to carcinoma in situ after a median of 48 months (Figure 4).

Univariate analysis for disease progression revealed a non-significant association of HIV-positive status and an increased risk of transformation, with an odds ratio (OR) of 2.79 (95% CI: 0.94–9.33, *p* = 0.073). Multivariable analysis did not reveal any factor significantly associated with the transformation of LSILs into HSILs or carcinoma in situ. Neither MSM (OR 0.98, 95% CI: 0.31–3.03, *p* = 0.970) nor the male sex (OR 0.75, 95% CI: 0.25–2.51, *p* = 0.616) were retained as risk factors.

### 3.4. Algorithm

**Figure 5:**
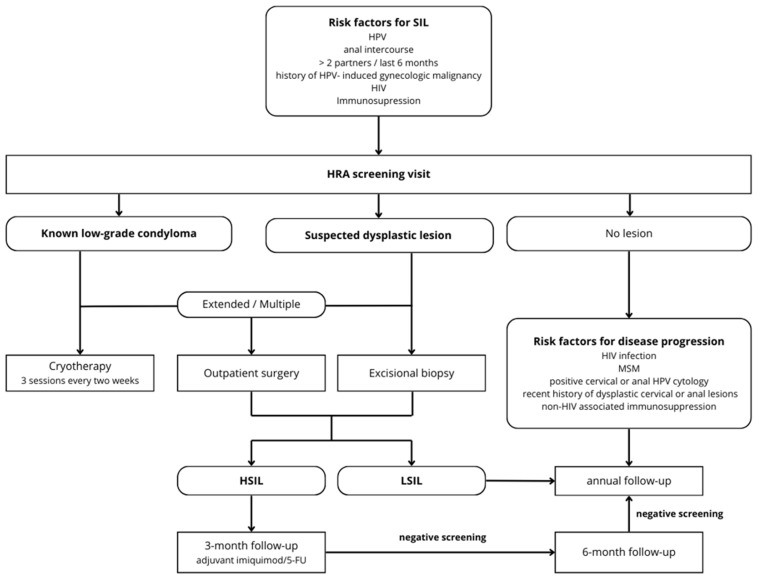
Institutional algorithm for HRA follow-up and treatment. HRA—high-resolution anoscopy, HPV—human papillomavirus, HIV—human immunodeficiency virus, HSIL—high-grade squamous intraepithelial lesion, LSIL—low-grade squamous intraepithelial lesion.

As a result of the above findings, our algorithm for a standardized management and surveillance strategy in the HRA clinic was adapted as detailed (Figure 5).

## 4. Discussion

The present study was conducted 10 years after the implementation of a specialized HRA-based outpatient clinic to evaluate institutional screening and treatment modalities, the efficacy of surveillance, and oncological outcomes. This longitudinal analysis revealed several important findings. First, over half of the cohort presented with (mainly low-grade) dysplastic lesions during follow-up. Risk factors for high-risk dysplasia were multi-fold and included the presence of HR-HPV genotypes, symptoms during the initial screening visit, and HIV infection (with detectable viral load). However, only in 6% of patients was disease progression to higher-stage lesions observed during the median follow-up period of 51 months, with 3% of patients progressing from HSILs to in situ carcinoma. While no patient progressed to invasive cancer, SCC was diagnosed in four patients during the initial screening visit.

Anal cancer represents only 6% of anorectal cancers and is nowadays equally distributed among men and women [7]. During the AIDS epidemic in the early eighties, a dramatic increase among the MSM population was observed, and the prevalence of HPV infection among HIV + MSM reached 100% [4]. Nowadays, anal cancer incidence remains 10–100x higher in immunosuppressed patients, such as transplant patients. Today, incidence rates range from 60 to 160 per 1,000,000 person–years for MSM people living with HIV, 32 for non-MSM HIV-positive men, and 9 to 48 per 1,000,000 person–years for females with HPV-related gynecological precancerous or cancerous lesions compared to the general population [14,20,21]. Of note, a recent study of our group revealed a 4-fold increased risk of anal high-risk HPV (HR-HPV) infection in women with cervical HR-HPV infection, emphasizing the importance of follow-up in sexually active women while bearing in mind the importance of concomitant cervical and anal screening [22]. Importantly, up to 80% of sexually active adults will contract anogenital HPV at some point in their life [15], with HPV remaining the main causative factor for LSIL and HSIL progression [16,23,24,25,26]. While HPV type 16 represents 73% of high-risk HPV strains, multiple strains are typically detected on anal swabs. Decreased immunity, including altered intracellular control, paracrine effects, and chromosomal instability, represents triggers of the HPV-promoted dysplasia to cancer sequence [12]. Sexual risk behaviors further enhance exposure, such as a notable number of sexual partners or non-protected anorectal intercourse [15]. Given that HPV persists within carriers over years, surveillance of risk populations is key for disease prevention [27].

The present study revealed several risk associations for ≥HSILs. The main risk factor yielding a 5-fold increased risk was related to the presence of HR-HPV genotypes, further highlighting the oncogenic potential in particular of types 16 and 18 [28]. The association of HIV infection and detectable viral load and higher-stage lesions has been repeatedly established. A landmark epidemiologic analysis evaluating this association in the male population in HIV-positive MSM emphasized the importance of the specific targeting of HPV 16-positive HSILs [15]. Symptoms related to the presence of perianal lesions such as severe discomfort, itching, or burning or bleeding on contact may indicate more advanced disease stages, simply reflecting the local extent of the disease [29]. However, these are unspecific to condyloma-like lesions; hence, this association should be interpreted with caution.

Interestingly, the present study further observed a preponderance of higher-stage lesions in female patients (as a statistical trend). A recent epidemiologic study computing age-standardized incidence rates of anal cancer found a most significant increase over the years in Swiss women among European countries [5]. The reasons for this finding could not be established; however, the analysis of our cohort appears to confirm this worrisome trend. Of note, the differences observed between the two multivariable analyses likely reflect the distinct populations and endpoints studied. The first analysis identified risk factors for the presence of advanced precancerous lesions (≥HSILs) in the overall cohort, whereas the second focused on the progression of LSILs to HSILs or carcinoma in situ. This distinction may explain why factors such as sex and MSM status, which are associated with the presence of lesions, do not appear as risk factors in the context of disease progression. Additionally, the small sample size in the progression analysis could limit the power to detect significant associations, which must be considered when interpreting the results. Of note, the statistical trend to higher-stage lesions in patients who were lost to follow-up before presenting to our clinic further highlights the importance of compliance to standardized surveillance schemes.

The prevalence of dysplastic lesions in this study was high, with almost half of the patients presenting with such lesions during the study period. Most institutional and regional care providers were aware of the specialized outpatient clinic at the CHUV, with referrals being motivated by the presence of suspicious lesions or specific risk profiles. In this setting, both the high prevalence and low progression rate align well with similar reports out of specialized clinics [30,31]. Interestingly, it was further demonstrated that detection rates for anal dysplasia were higher when performed in the operating room [32]. Taken together, the high prevalence in the present cohort confirms the importance of risk-targeted screening, as advocated by the most recent international guidelines of various high-risk groups [33,34].

In the setting of the present longitudinal analysis, we closely monitored disease progression not only in patients with HIV infection and immunosuppression but also in MSM and cervical HPV carriers. Our findings of a very low progression rate even in these risk groups align well with the results of a former 10-year series, as well as a landmark meta-analysis revealing an estimated progression rate to invasive carcinoma of 1.2% [35,36]. Our results reiterated a three-fold increased risk of disease progression to higher-stage lesions in HIV-positive patients; however, due to the low progression rate, the analysis of risk factors was limited and must be interpreted with caution. Larger cohorts are needed to confirm our findings and further establish patterns of disease progression in all-comers.

Specialized follow-up units are mandatory to reliably screen and appropriately treat populations at risk. HRA is now considered the gold standard in the multi-staged screening protocol of anal carcinoma, including digital anorectal examination, cytology, and HPV testing [37,38,39]. While several international oncological and surgical societies have yet to adopt a standardized screening protocol [23,24,40,41,42], a multitude of screening recommendations and guidelines are available [43]. The International Anal Neoplasia Society (IANS) developed consensus guidelines for anal cancer screening among various high-risk groups [33]. Guided by risk thresholds, screening initiation at age 35 years was recommended for men who have sex with men (MSM) with HIV. For other people with HIV and MSM without HIV, screening initiation at age 45 years was recommended. For solid organ transplant recipients, screening initiation beginning from 10 years post-transplant was recommended. Finally, for persons with a history of vulvar precancer or cancer, screening initiation was recommended to start within 1 year of diagnosis of vulvar precancer or cancer. Anal cytology, HR-HPV testing (including genotyping for HPV16), and HR-HPV–cytology co-testing are different strategies currently used and recommended for anal cancer screening, with HRA or follow-up screening tests if warranted [33]. Our group recently adapted the screening protocol for HR-HPV-positive women, with the recommendation of systematically performing anal cytology given a 4-fold increased risk of concomitant infection [22]. The present study further allowed us to refine the institutional screening algorithm in light of these recommendations (Figure 5).

Invasive anal carcinoma remains a rare disease in the general population but has a substantial impact on functional outcomes and quality of life (QoL), both after radiotherapy (continence issues) and surgery (abdominoperineal resection with lifetime end colostomy in non-responders). Standardized screening strategies for high-risk populations are hence mandatory, considering that anal carcinoma incidence rose at least 2.2% per year in the past decade (up to 8.6% in men and 7.5% in women for advanced stages) [7]. Today, patients with anal carcinoma have a 5-year survival rate of 30% for metastatic disease and an annual mortality rate exceeding 3% per year [43]. Since progression from dysplastic lesions to invasive anal carcinoma is slow, early detection and intervention are warranted, given the encouraging survival rate of over 80% for localized stages [7].

Optimal screening protocols should also include a coordinated vaccination campaign, with a potential to substantially impact the natural history of the disease, as previously observed for cervical carcinoma. A Swedish population-based study observed a drastic drop in the cumulative incidence of cervical cancer from 94 to 47 cases per 100,000 persons after the implementation of a nationwide quadrivalent HPV vaccination [44]. Current guidelines suggest the vaccination of young females (9–26 years old) and males (9–15 years old). The nine-valent vaccine offers protection against aggressive (types 16 and 18) and frequent lesion-associated variants (types 6, 11, 31, 33, 45, 52, 58) amongst the 200 reported HPV genotypes [45,46]. The vaccine is most effective when administered at a young age and before all sexual activity in HPV-naive individuals. In the presence of pre-existing dysplasia, these protocols may lead to a decreased risk of new HPV-linked lesions, potentially reducing the incidence of anal carcinoma [16,47,48,49].

This study has several limitations. First, the retrospective design and single-center setting may limit the generalizability of our findings. Loss to follow-up could have led to an underestimated progression rate, particularly for patients with more severe disease. Furthermore, the absence of a control group impedes direct comparison with alternative screening strategies. Second, patients were stratified to high- and low-risk HPV genotypes for the purpose of this study, with detailed representation of preponderant types 6, 11, 16, and 18. Further subgroup analysis of less frequent HR- and LR-HPV genotypes was not performed. The algorithm was designed to guide clinical decision-making based on cytological and histological findings rather than detailed genotyping data. Incorporating this level of analysis was not feasible within the operational constraints of the outpatient setting. Instead, our emphasis was on implementing a practical, scalable screening approach that could be readily applied in similar clinical environments outside of highly specialized settings. Third, the results of this longitudinal analysis are representative of a clinical practice dealing with all patients from a multitude of referring departments or physicians and may hence not be comparable to a highly specialized setting (i.e., AIDS clinic). Hence, these findings cannot be uncritically extrapolated to other settings or institutions. Future research protocols, ideally within a prospective or even randomized controlled setting, should further evaluate the effectiveness of screening protocols in diverse populations to refine anal cancer prevention strategies.

## 5. Conclusions

The decisive role of HPV infection, in particular related to high-risk genotypes, in the pathogenesis of anal cancer makes it a largely preventable disease. The present analysis 10 years after the implementation of a dedicated HRA clinic revealed the efficient detection and management of dysplastic anal lesions in high-risk populations with a low progression rate to higher-stage lesions and allowed us to refine the institutional screening and treatment schemes. Given the rising incidence and substantial mortality of anal SCC, the implementation of standardized screening strategies and expanding HPV vaccination in risk groups are essential for the prevention of disease progression to cancer.

## Figures and Tables

**Table 1:** Patient’s baseline demographics and clinical variables.

Item	All Patients (n = 537)	LSIL (n = 181)	≥HSIL (n = 82)	No SIL (n = 274)	*p*-Value
Age (median, IQR)	47 (38–57)	44 (36–53)	53 (41–64)	47 (39–57)	<0.001
Male gender (%)	377 (70.2%)	139 (77.8%)	46 (58.2%)	194 (68.8%)	0.001
MSM (% of men)	265 (70.3%)	90 (64.7%)	28 (60.1%)	147 (75.8%)	0.01
HPV related symptoms (%)	114 (21.2%)	59 (32.6%)	25 (30.5%)	30 (10.9%)	<0.001
Immunosuppression					
HIV-related (%)	280 (52.1%)	70 (39.7%)	32 (40.5%)	178 (63.1%)	<0.001
Non-HIV-related (%)	18 (3.4%)	6 (3.4%)	(6.3%)	7 (2.5%)	0.05
Detectable HIV viral load (%)	40 (7.4%)	9 (5.0%)	10 (12.2%)	21 (7.7%)	0.03
AIDS (A3, B3, C3)	90 (16.8%)	26 (14.4%)	16 (19.5%)	48 (17.5%)	0.52
Charlson Comorbidity Index ≥ 2	183 (34.1%)	53 (29.3%)	37 (45.1%)	93 (33.9%)	0.05
Condyloma					
Endoanal (%)	193 (35.9%)	105 (58.0%)	59 (72.0%)	29 (10.6%)	<0.001
Perianal (%)	217 (40.4%)	124 (68.5%)	48 (58.5%)	45 (16.4%)	<0.001
Prior treatment					
Yes (%)	206 (38.4%)	80 (40.2%)	43 (52.4%)	83 (30.3%)	<0.001
Surgical excision (%)	128 (28.9%)	41 (22.7%)	29 (35.4%)	58 (21.2%)	0.02
Cryotherapy (%)	41 (7.63%)	22 (12.2%)	5 (6.1%)	14 (5.1%)	0.02
Laser (%)	25 (4.65%)	11 (6.1%)	4 (4.9%)	10 (3.6%)	0.49
Local therapy (%)	82 (15.3%)	40 (22.1%)	8 (9.8%)	34 (12.4%)	0.01
Prior HPV genotyping					
HR-HPV (%)	64 (11.9%)	15 (8.3%)	15 (18.3%)	34 (12.4%)	<0.001
HPV-16	27 (5.0%)	5 (2.8%)	13 (15.9%)	9 (3.3%)	
HPV-18	7 (1.3%)	1 (0.6%)	2 (2.4%)	4 (1.5%)	
Other HR types	48 (8.9%)	11 (6.1%)	10 (2.2%)	27 (9.9%)	
LR-HPV (%)	36 (6.7%)	10 (5.5%)	8 (9.8%)	18 (6.6%)	0.44
HPV-6	14 (2.6%)	7 (3.9%)	3 (3.7%)	4 (1.6%)	
HPV-11	6 (1.1%)	2 (1.1%)	2 (2.4%)	2 (0.7%)	
Other LR types	24 (4.5%)	4 (2.2%)	5 (6.1%)	15 (5.5%)	
HPV genotyping					
HR-HPV (%)	111 (20.7%)	57 (31.5%)	47 (57.3%)	7 (2.6%)	<0.001
HPV-16	38 (7.1%)	11 (6.1%)	27 (32.9%)	0 (0.0%)	
HPV-18	18 (3.4%)	6 (3.3%)	11 (13.4%)	1 (0.4%)	
Other HR types	86 (16.0%)	48 (26.5%)	31 (37.8%)	7 (2.6%)	
LR-HPV (%)	169 (31.5%)	123 (68.0%)	31 (37.8%)	15 (5.5%)	<0.001
HPV-6	107 (19.9%)	81 (44.8%)	14 (17.1%)	12 (4.4%)	
HPV-11	42 (7.8%)	29 (16.0%)	11 (13.4%)	2 (0.7%)	
Other LR types	57 (10.6%)	37 (20.4%)	17 (20.7%)	3 (1.1%)	

LSIL—low-grade squamous intraepithelial lesion, HSIL—high-grade squamous intraepithelial lesion, SIL—squamous intraepithelial lesion, ≥HSIL: cumulative population presenting with HSILs, carcinoma, in situ, or cancerous lesions. HPV—human papilloma virus. Results of HPV genotyping correspond to the number of patients who tested positive for a specific genotype. One patient can account for several groups due to cooccurrence.

## Data Availability

The data presented in this study are available on request from the corresponding author. The data are not publicly available due to ethical committee restrictions.

## Abbreviations

HRA—high-resolution anoscopy, HPV—human papillomavirus, HIV—human immunodeficiency virus, HSIL—high-grade squamous intraepithelial lesion, LSIL—low-grade squamous intraepithelial lesion, MSM—men who have sex with men, SCC—squamous cell carcinoma.
